# Bioinformatics analysis-based screening of circRNA gene with mainstream expression trend in colorectal cancer and construction of a coexpression regulatory network

**DOI:** 10.1371/journal.pone.0295126

**Published:** 2023-12-08

**Authors:** Lei Xu, Hongqiang Zhang, Yu Shao, Zan Fu

**Affiliations:** Department of General Surgery, The First Affiliated Hospital of Nanjing Medical University, Nanjing, Jiangsu, China; University of the Punjab, PAKISTAN

## Abstract

**Objective:**

Since circRNA can be utilized as a potential diagnostic marker for cancer, to explore the regulatory mechanism of colorectal cancer (CRC) using bioinformatics, the public database of circRNA was mined.

**Methods:**

CRC differentially expressed miRNAs were screened in the Cancer Genome Atlas (TCGA) database, CRC differentially expressed circRNAs were searched in the Gene Expression Omnibus (GEO) database, the two databases were combined to identify CRC differentially expressed mRNAs, and a circRNA-miRNA‒mRNA regulatory network was constructed by combining a plurality of target prediction databases to identify key genes. The upstream circRNA and regulatory axis of the key genes were identified for gene ontology (GO) and Kyoto encyclopedia of genes and genomes (KEGG) enrichment analysis to explore the biological functions of circRNA in CRC using the regulatory axis.

**Results:**

After the screening of the GSE21815 dataset, a total of 22 differentially expressed circRNAs were obtained, with 12 upregulated and 10 downregulated genes. Similarly, the GSE126094 dataset yielded 104 differentially expressed circRNAs, comprising 56 upregulated and 48 downregulated genes. Among the differentially expressed circRNAs, five were identified, with VDAC3 and SETD2 showing downregulated expression, while RAD23B, RPPH1, and MYBL2 exhibited upregulated expression. Following the selection process, five DEcircRNAs, eight target miRNAs, and 105 target DEmRNAs were identified. The protein-protein interaction (PPI) network revealed close relationships among the mRNAs, with E2F2, E2F3, CCND1, TNRC6A, and KAT2B identified as key genes. Notably, CCND1 emerged as a critical gene in the PPI network. Through the upregulation of has-circ-0087862, which binds to miR-892b, the translation inhibition of CCND1 by miR-892b was attenuated, leading to enhanced CCND1 expression. Functional enrichment analysis indicated that CCND1 was involved in protein binding and positive regulation of cellular processes, among other functions.

**Conclusion:**

The differentially expressed genes (DEGs) in CRC markedly affected the survival time of patients. CircRNAs could be utilized as diagnostic markers of CRC, and the key genes in CRC could be screened out by bioinformatics, which would be helpful to understand the drug targets for the treatment of human immunodeficiency virus (HIV)-related CRC patients.

## 1. Introduction

Colorectal cancer (CRC) is a common large digestive malignant tumor, with an incidence rate ranking third among all tumors [1]. CRC accounts for approximately 10% of the global annual cancer diagnosis rate and cancer-related mortality rate [2]. According to malignant tumor data from China in 2015, the number of new cases of CRC was approximately 376,000, and the annual death toll was approximately 191,000. According to the statistics of the American Cancer Society [3], recent studies have indicated that intestinal flora imbalance, the production of bacterial toxins, the impact of carcinogenic metabolism, and the promotion of the inflammatory response are involved in the occurrence of CRC [4]. Numerous scholars have conducted research on the pathogenic mechanisms of CRC, which has provided new insights for diagnosis and treatment. Zhu et al. (2019) [5] utilized the publicly available datasets GSE21510 and GSE32323 to screen for differentially expressed genes (DEGs), revealing CDKN2B, CDKN1A, MYC, SKP2, and E2F2 as core genes associated with CRC. Cui et al. (2017) [6] suggested that DEPTOR, AURKA, CCND1, BCAS1, NEDD9, and MAP2K2 may play a role in CRC. Qi et al. (2018) [7] confirmed the upregulation of EGFR, HRas, and Akt1 as DEGs, while Wnt5a and CDKN1a were found to be downregulated DEGs. Yu et al. (2019) [8] proposed that TOP2A, MAD2L1, CDC6, and CHEK1 could serve as prognostic biomarkers for CRC. The pathogenesis of CRC is relatively complex, involving interactions between multiple pathways and multiple genes. Different scholars have presented varying core genes associated with CRC, indicating that the underlying molecular mechanisms and key genes involved in CRC metastasis are still not fully elucidated.

Transcriptional profiling techniques, such as transcriptome sequencing and expression microarray technology, have been widely employed to identify DEGs. These technologies enable the recognition of transcriptional differences across different pathological states, the discovery of novel biomarkers, and the provision of valuable insights into disease progression [9]. Both exons and introns can form circRNA, which has no typical terminal structure and is resistant to nucleic acid exonuclease. This is the reason for the high stability of circRNA. Research in the new phase [10] indicates that the roles of circRNA in cancer include cancer diagnosis, prognostic markers, competitive binding of miRNA, methylation modification, influence on transcription and splicing, and regulation of drug resistance. MicroRNAs can induce gene silencing by binding to mRNA, while competing endogenous RNAs (ceRNAs) regulate gene expression by competitively binding to microRNAs. The ceRNAs can play a significant biological role by sequestering microRNAs through their microRNA response elements (MREs), leading to the inactivation of microRNAs. In all organisms, circRNAs compete for binding with microRNAs, which can result in the degradation or translational inhibition of target mRNAs, depending on the complementarity between miRNA and mRNA sequences. In plants, miRNAs and their target mRNAs exhibit complementarity, whereas in animals, most miRNAs do not show complete complementarity with their target mRNAs. Therefore, miRNAs primarily regulate the expression of target mRNAs through translational inhibition [11]. Bioinformatics has been used by some scholars in the research of CRC inflammatory diseases, providing candidate genes for the occurrence of inflammation-related rectal cancer [12]. Since Li et al. (2021) [13] proposed the multigene and multistage CRC occurrence mode, great progress has been made in CRC-related molecular biology. The molecular mechanism of CRC occurrence is the clearest among solid tumors, but the diagnosis and treatment of CRC in the progressive stage have not made breakthrough progress to date [14]. Bioinformatics is also database mining, with the advantages of a large sample size, low cost, simplicity, and efficiency, using different databases to obtain a large amount of data, developing relevant algorithms and software, integrating and comparing the mechanisms of biological development, and identifying the relevant biomarkers, which has become a way to explore disease gene mutation [15–17]. The effective mining and development of gene expression of related diseases using public databases makes it possible to study biological life acquisition and disease occurrence using intelligent systems, which has a profound impact on the prevention and treatment of diseases [18]. At present, there is a lack of ideal early diagnostic biomarkers for CRC. Given the biological characteristics, circRNAs are suitable candidates as diagnostic markers for CRC. However, the current research on circRNAs in CRC is still in its early stages [19].

Therefore, in the present study, circRNAs differentially expressed in CRC were searched from the Gene Expression Omnibus (GEO) database, and their diagnostic value was analyzed. Then, a protein‒protein interaction (PPI) network was established, key genes were identified, a circRNA regulatory network of key genes was fabricated, and gene ontology (GO) and Kyoto encyclopedia of genes and genomes (KEGG) enrichment analyses were implemented. Efforts are being made to explore the association between circRNAs and CRC, identify potential mechanisms, and provide insights for clinical treatment of CRC.

## 2. Materials and methodologies

### 2.1 Data source

The databases utilized in this study are publicly available and free of charge. GEO is currently the largest and most complete gene expression database established by the National Biotechnology Information Center of the United States in 2000. The database (https://www.ncbi.nlm.nih.gov/geo/) contained data on multiple gene expression of circRNA. In March 2020, it included 3,481,605 samples, 126,260 series, and 20,651 platforms. The GEO database includes Platform (GPL), Sample (GSM), and Series (GSM). Series (GSM) is a data set that combines all the sample information of an experiment. Sample (GSM) mainly records the conditions for processing a single sample and the basic information of the sample. The Platform (GPL) contains the sequence and a brief description of the array platform. The overexpression of circRNA in GEO database samples was associated with age, and samples with large age differences were excluded from the study. The microarray data sets GSE126094 and GSE142837 (species: *Homo sapiens)* were downloaded from the public database, and gene expression was detected using the GPL6244 detection platform and Affymetrix Gene Chips Human Gene 1.0ST chip. GSE21815 consisted of 9 normal controls and 132 CRC patients using the human genome 4×44KG4112F chip GPL6480 platform (Agilent, America). GSE126094 is a dataset with a chip platform GPL19978, consisting of expression profiles of circRNAs in 10 CRC tissues and their corresponding normal tissues.

To acquire genes related to CRC, a total of 480 CRC tumor tissue samples and 41 adjacent normal tissue samples were obtained from The Cancer Genome Atlas (TCGA) data portal (https://cancergenome.nih.gov). Gene expression profiles were downloaded for mRNA from these samples. Genes with incomplete clinical information were excluded from the analysis. Based on the survival time of CRC patients, only clinical information from patients with a survival time greater than 30 days was retained, resulting in a total of 462 samples.

GTEx (https://gtexportal.org/) is a database supported by various institutions, including the National Institutes of Health, that provides gene expression data from 44 different tissues of 449 healthy individuals. Since the number of normal samples in TCGA is relatively small, it is advisable to combine the analysis of mRNA data with GTEx for a comprehensive study.

The GSE126094 and GSE21815 data sets were obtained using the GEO query package in R language, and then gene name annotation was performed on the gene chip probes of the data set using the hugene10sttranscriptcluster.db package to generate gene expression values and gene names. Due to the presence of variant genes considered as background noise, gene screening was performed based on variance. The top 25% of genes with higher variance were selected for subsequent analysis. Pearson correlation matrix and clustering of different samples were utilized to remove samples with inadequate chip performance.

Key genes were identified using the CytoHubba plugin. Hub genes, also known as central genes, refer to genes that act as bottleneck genes connecting multiple nodes in a network. Key genes were selected from the pool of hub genes based on higher significant P-values. Core genes, on the other hand, are genes that are shared among different species in terms of biological diversity. The Venny2.1 tool (http://bioinfogp.cnb.csic.es/tools/venny/) was employed to produce a Venn diagram.

Sangerbox (http://sangerbox.com/index.html) is a software developed by Hangzhou Muguo Technology Co., Ltd. It integrates tools such as GEO data and Impact Factor (IF) conversion, as well as volcano plot generation, to facilitate the search for circRNA series in the GEO database.

The DECenter differential analysis tool is based on the R language and the limma package. It is used to identify differentially expressed circRNAs between cancer and adjacent normal tissues in an expression matrix. The ID conversion tool allows for the retrieval of circRNA expression from MINIML formatted files downloaded from the GEO database. It enables the extraction of expression levels for each circRNA in each sample and facilitates the conversion of probe IDs to circRNA IDs using the Affymetrix equation, resulting in a series matrix.

### 2.2 STING database

The search tool for the retrieval of interacting genes (STRING) database (http://string-db.org/) is a repository of PPI data. The screened protein names corresponding to the differential genes were input into STRING so that an interaction network between coded proteins can be obtained in the STRING database, and the connectivity of each differential protein can be calculated. The MCODE plugin in Cytoscape software was used to identify a subset of genes with the most closely interconnected relationships in a PPI network. Among these genes, the one with the highest degree was considered a key gene.

### 2.3 Functional enrichment analysis

GO analysis, which consists of Cellular Component (CC), Molecular Function (MF), and Biological Process (BP) categories, helps elucidate the biological functions of specific genes from different perspectives. KEGG analysis allows for the identification of biological pathways in which certain genes are enriched. Using GO enrichment analysis, this information can be utilized to identify biological processes, molecular functions, and cellular components and to extract genes with the highest and lowest values from gene expression profiles to produce upregulated and downregulated genomes.

### 2.4 Identification of differentially expressed circRNAs

Sangerbox (http://vip.sangerbox.com) was utilized to find the differentially expressed circRNAs in the circRNA series in the GEO database. Sanger Box V 1.0.9 was developed by Hangzhou Mogu Technology Co., Ltd., which can convert GEO data ID. The text of circRNA chip series expression data MINiML formatted family file was downloaded in GEO, from which the expression amount of each CIRCRNA in each sample was extracted and converted to obtain the expression matrix of the modified series.

The search was conducted using the keywords “CRC” and “circRNA”. The species “Human” was screened to download the microarray data set in the public database, and the research types selected were “noncoding RNA profiling by array” and “noncoding RNA profiling by high throughput sequencing” to screen cancer and cancer-adjacent tissue samples. GSE126094 and GSE21815 were selected from two data sets to identify DEGs in the CRC data sets.

### 2.5 Western blot analysis of Ncadherin and Ecadherin protein expression

Total protein was extracted from cells by radioimmunoassay buffer. After analyzing the protein concentration, 30μg of protein sample was loaded on sodium dodecyl sulfate polyacrylamide gel electrophoresis gel and separated, and then the sample was transferred to polyvinylidene fluoride membrane. The membrane was sealed in 5% skimmed milk powder for 1h at room temperature, and then stayed overnight at 4°C with primary antibodies: Ncadherin antibody (1:800), Ecadherin antibody (1:500) and internal reference GAPDH antibody (1:1000). On the second day, the membrane was incubated with the secondary antibody coupled with horseradish peroxidase (HRP) for 2 hours at room temperature. The color reaction was carried out with chemiluminescence reagent, and the expression of target protein was analyzed with Image J.

### 2.6 Statistical analysis

Employing SPSS 18.0, statistical analysis and processing of the data were implemented. When *P*<0.05, the difference was statistically significant. Quantitative data were recorded in the form of mean plus or minus standard deviation, and categorical data as percentages. Differences between groups were tested using a chi-square test, paired sample Student’‘s *t* test, independent sample *t* test, and repeated measures analysis of variance (where applicable). The significance level of the bilateral test was set at *P*< = 0.05.

## 3. Results

### 3.1 GSE126094 database patient information

The general information of the 10 patients in the GSE126094 data is shown in Table 1. There were six males and four females, and they were aged from 56 to 74 years old, with an average age of 64.9±9.2 years old.

**Table 1:** Paracancerous tissue sample information for patients in data set GSE126094.

No.	Sex	Age	Paracancerous sample	Cancer sample
**1**	Female	60	GSM3591565	GSM3591546
**2**	Male	56	GSM3591564	GSM3591547
**3**	Male	66	GSM3591563	GSM3591548
**4**	Female	74	GSM3591562	GSM3591549
**5**	Male	59	GSM3591561	GSM3591550
**6**	Male	61	GSM3591560	GSM3591551
**7**	Female	61	GSM3591559	GSM3591552
**8**	Female	67	GSM3591558	GSM3591553
**9**	Male	72	GSM3591557	GSM3591554
**10**	Male	65	GSM3591556	GSM3591555

### 3.2 Venn analysis of DEGs in two data sets

**Fig 1:**
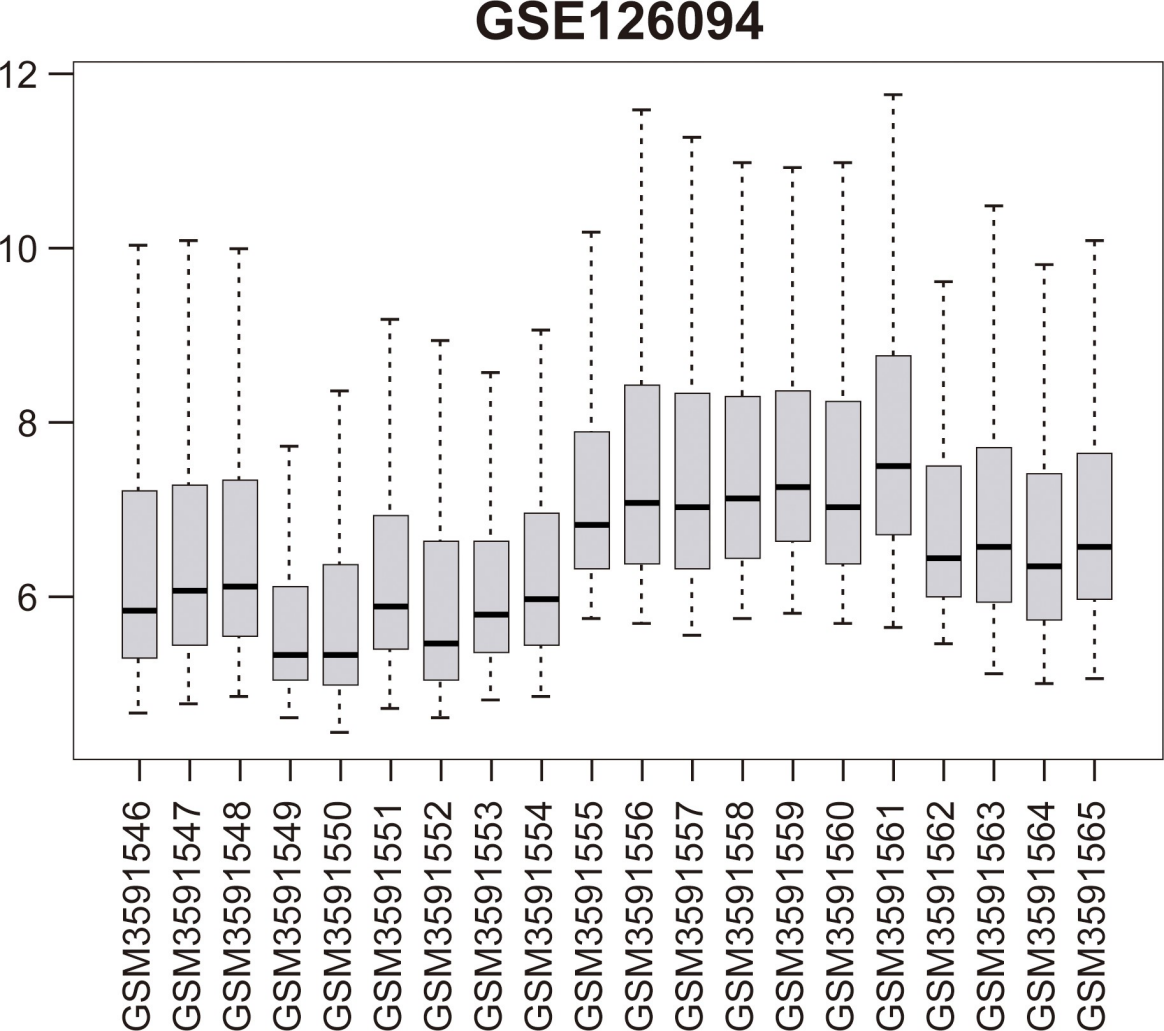
Visualization diagram of GSE126094 dataset.

**Fig 2:**
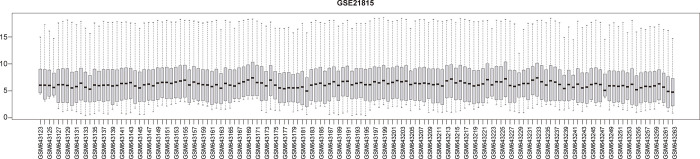
Visualization diagram of GSE21815 dataset.

**Fig 3:**
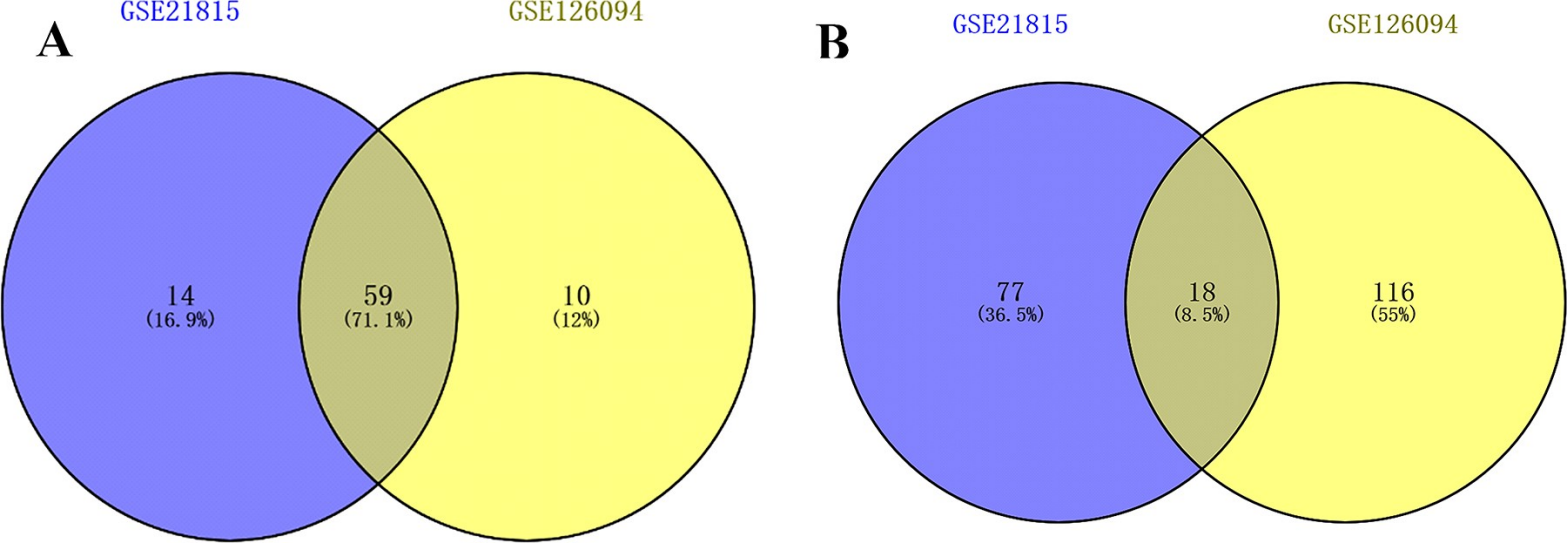
Venn diagrams visualizing the differentially upregulated and downregulated genes in the two datasets. (Note: A represents upregulated genes, and B represents downregulated genes).

Two Venn diagrams were constructed for the two datasets (Figs 1 and 2), resulting in 59 upregulated genes (Fig 3A) and 18 downregulated genes (Fig 3B), respectively.

### 3.3 Differentially expressed circRNA

**Fig 4:**
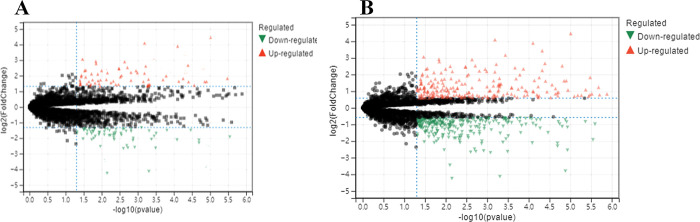
A: volcano map of GSE 21815; B: volcano map of GSE126094.

After the visualization data was obtained from the GSE21815 and GSE126094 datasets, the Sangerbox software was utilized to extract the expression matrices and convert the IDs. Subsequently, differential expression volcano plots were generated for circRNAs. In the GSE21815 dataset, there were a total of 2,143 circRNAs. The filtering criteria used were |log_2_FC| > 1.5 and adj.P < 0.01. As a result, 22 differentially expressed circRNAs were identified, including 12 upregulated and 10 downregulated circRNAs. In the GSE126094 dataset, there were a total of 4012 circRNAs. Using the same filtering criteria, 104 differentially expressed circRNAs were obtained, including 56 upregulated and 48 downregulated circRNAs (Fig 4).

**Fig 5:**
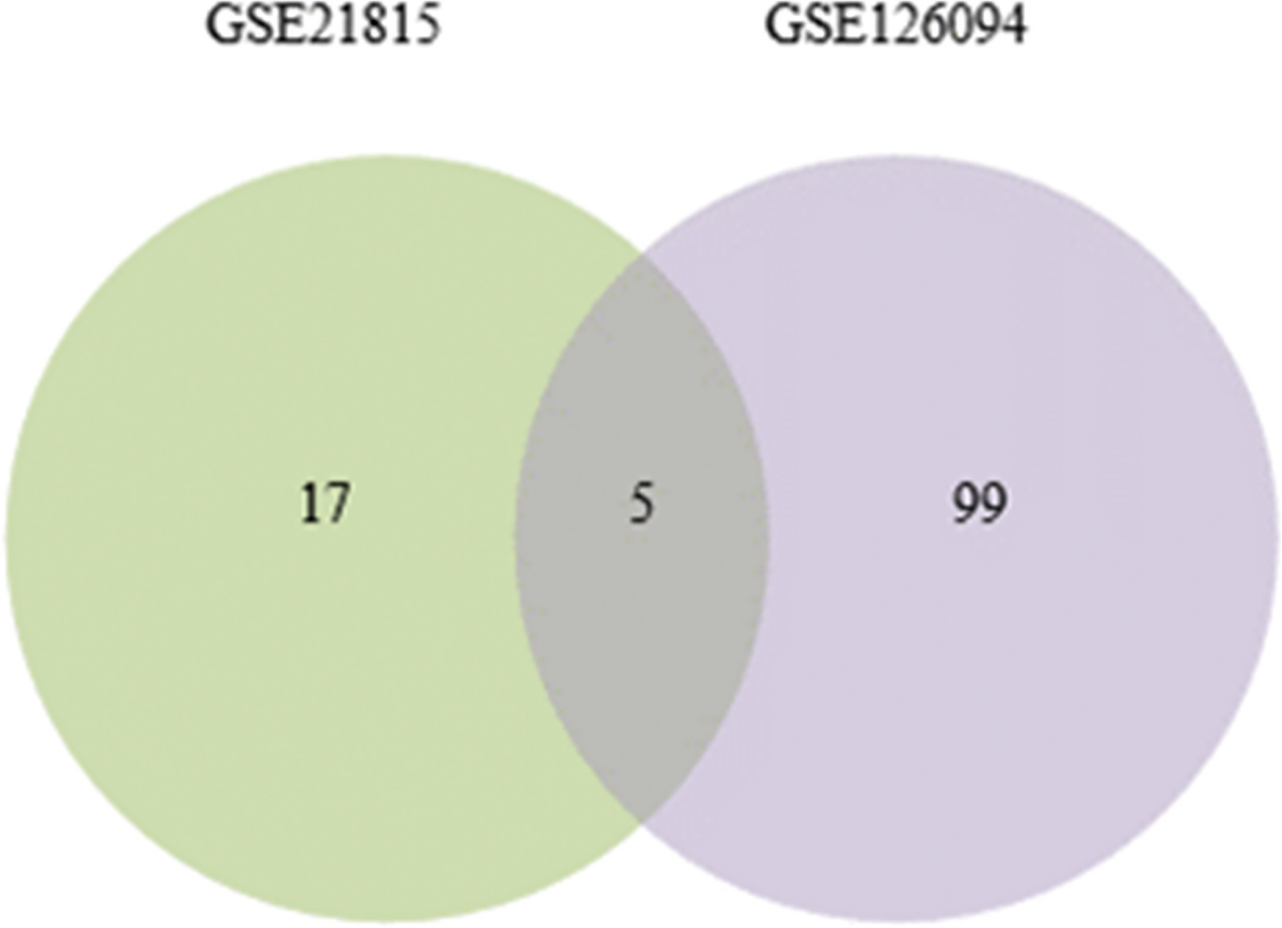
Venn diagram of differentially express circRNA.

The differentially expressed circRNAs from the GSE21815 and GSE126094 datasets were visualized as a Venn diagram (Fig 5). The circRNA host genes and sequence lengths were retrieved from the circBank database. In the GSE126094 dataset, which had a larger number of samples, the information included expression levels (upregulated or downregulated), |log_2_FC| values, and adj.P values.

The five differentially expressed circRNA are shown in Table 2, in which the expressions of VDAC3 and SETD2 genes were down-regulated, and the expressions of RAD23B, RPPH1, and MYBL2 genes were up-regulated.

**Table 2:** Differentially expressed circRNAs.

No.	Host gene	|log2FC|	Sequence length	circRNA	Expression	adj.P
**1**	VDAC3	2.126199	379	HAS-circ-0005927	Down-regulation	2.04E-03
**2**	SETD2	2.608276	4644	HAS-circ-0065173	Down-regulation	6.61E-06
**3**	RAD23B	2.702212	349	HAS-circ-0006174	Up-regulation	1.34E-03
**4**	RPPH1	2.622354	150	HAS-circ-0000518	Up-regulation	5.90E-06
**5**	MYBL2	1.581415	554	HAS-circ-0006332	Up-regulation	2.81E-06

### 3.4 Differentially expressed target miRNA

A total of 358 mature miRNA expression data for CRC were downloaded from the TCGA database using the Sangerbox tool (http://sangerbox.com/index.html). This dataset included 302 CRC samples and 46 normal samples. The miRNA encoding was converted to miRNAID, and the data were standardized to eliminate differences between samples. The differential analysis revealed a total of 2,217 miRNAs in the TCGA CRC dataset. Subsequently, using the criteria of |log_2_FC| > 1.5 and adj.P < 0.01, 427 differentially expressed miRNAs were identified, comprising 189 upregulated genes and 238 downregulated genes.

**Fig 6:**
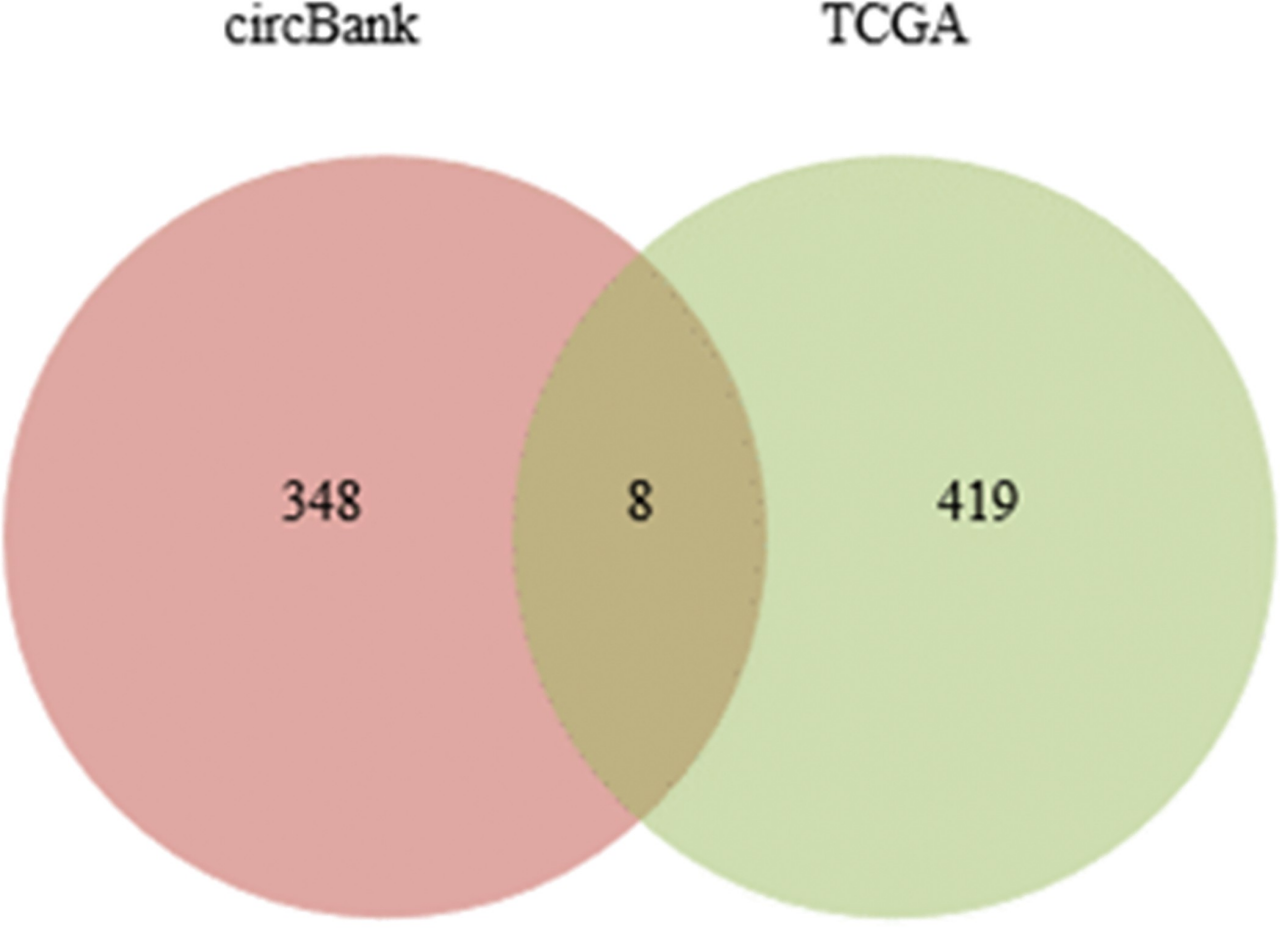
Venn diagram of differentially expressed target miRNA.

To predict the potential RNA targets for the five differentially expressed circRNAs, circBank database was employed, resulting in the identification of 356 potential targets. A Venn diagram was constructed to visualize the overlap between the differentially expressed miRNAs obtained from the TCGA database and the miRNAs predicted by circBank database (Fig 6).

Eight differentially expressed target miRNA are shown in Table 3.

**Table 3:** Eight differentially expressed target miRNA.

No.	miRNA	|log_2_FC|	Expression	adj.P
**1**	HAS-miR-577	5.917345	Up-regulation	3.74E-07
**2**	HAS-miR-217	3.88088	Up-regulation	1.54E-11
**3**	HAS-miR-127-5p	3.465434	Up-regulation	5.39E-33
**4**	HAS-miR-936	4.151551	Down-regulation	4.98E-05
**5**	HAS-miR-892b	3.127163	Down-regulation	8.21E-05
**6**	HAS-miR-1182	4.209639	Down-regulation	3.59E-05
**7**	HAS-miR-326	3.424992	Down-regulation	1.95E-30
**8**	HAS-miR-1270	2.438654	Down-regulation	1.86E-05

### 3.5 Differentially expressed mRNA

**Fig 7:**
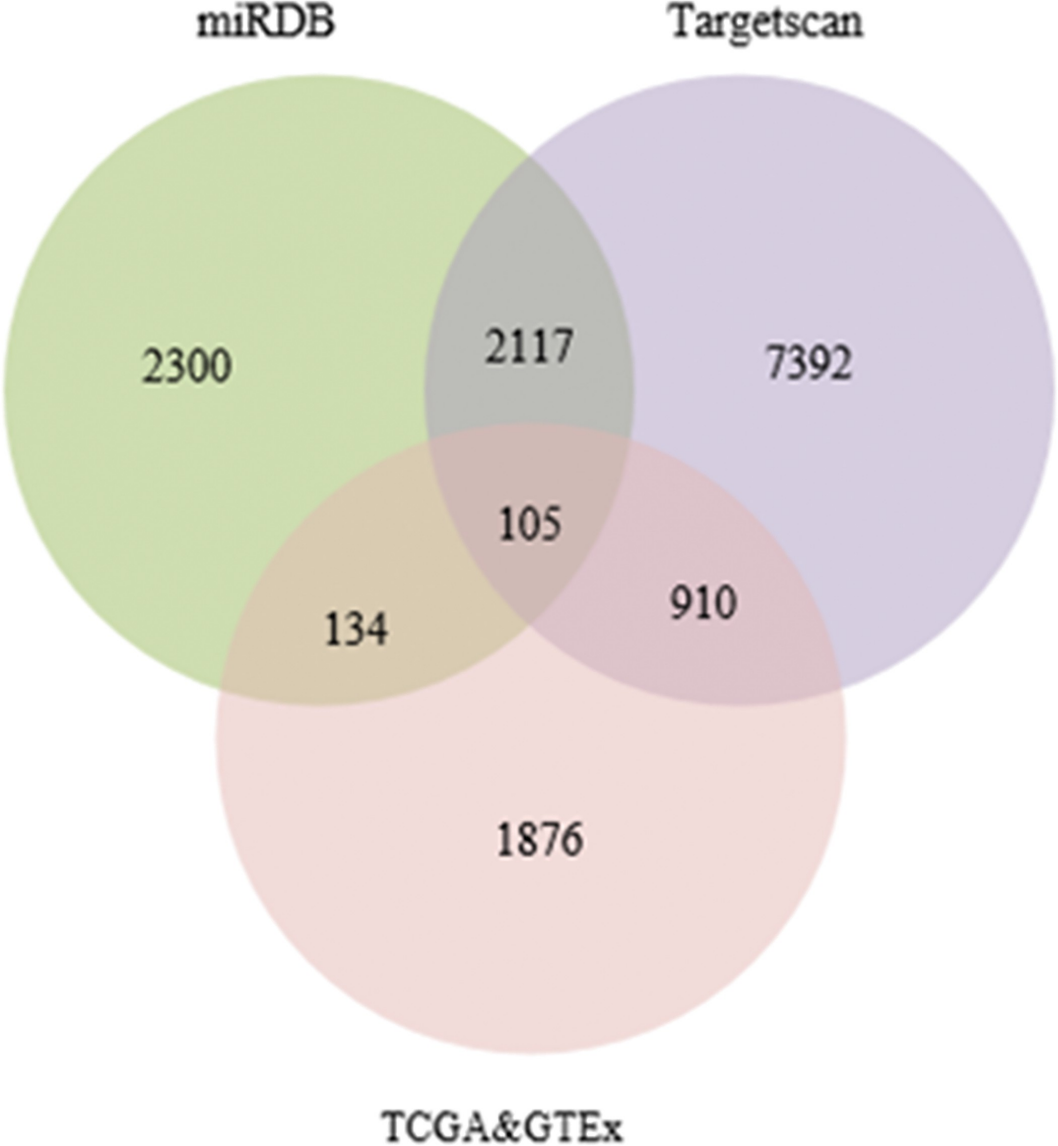
Venn diagram of differentially expressed target mRNA.

A total of 706 CRC samples were obtained from the GEPIA2 database, including 169 colon cancer samples, 50 rectal cancer samples, and 189 cancer samples. Among them, there were 298 normal samples. Using the criteria of |log_2_FC| > 1.5 and adj.P < 0.01, 4,367 differentially expressed mRNAs were identified in colon cancer, 3806 differentially expressed mRNAs in rectal cancer, and 3,025 commonly expressed mRNAs in CRC. Among the commonly expressed mRNAs, 1,587 were upregulated and 1,438 were downregulated. The miRDB and TargetScan databases were utilized to predict the potential mRNA targets of the 8 aforementioned differentially expressed miRNAs. The results yielded 4,656 and 10,524 potential target mRNAs, respectively. Additionally, by incorporating the differentially expressed mRNAs identified from the TCGA&GTEx databases, a Venn diagram was constructed, which revealed an intersection of 105 DEmRNAs (Fig 7).

### 3.6 PPI network diagram

After filtering, a total of 5 DEcircRNA, 8 target miRNA, and 105 target DEmRNA were obtained. Nine isolated mRNAs were removed from the intersection network of target interactions, resulting in a final set of 96 target DEmRNA, 5 DEcircRNA, and 8 target miRNAs.

**Fig 8:**
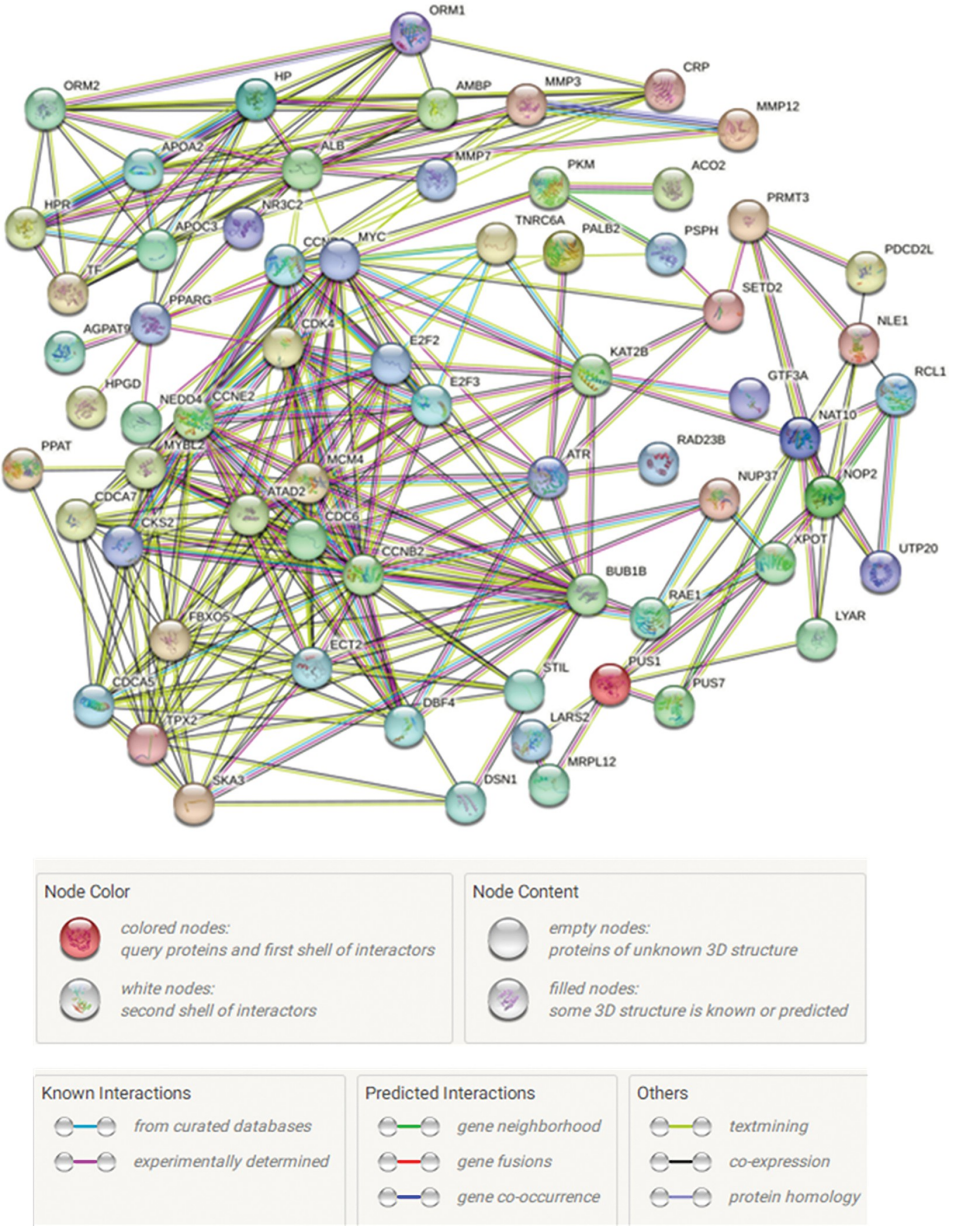
Protein interaction network diagram.

In the STRING database, the 96 mRNAs involved in the regulatory network were inputted into the search box of the Multiple Proteins module. The species selection was set as “human,” and the search results provided the initial PPI network for the DEG network. The results revealed a total of 288 edges, an average node degree of 8.11, an average clustering coefficient of 0.668, and an expected number of edges of 96. The PPI enrichment p-value was < 1.0e^-16^. The degree of each node represents the number of proteins it interacts with in the network, and the lines indicate the closeness between proteins. A higher number of lines indicates a greater association with related genes (Fig 8).

**Fig 9:**
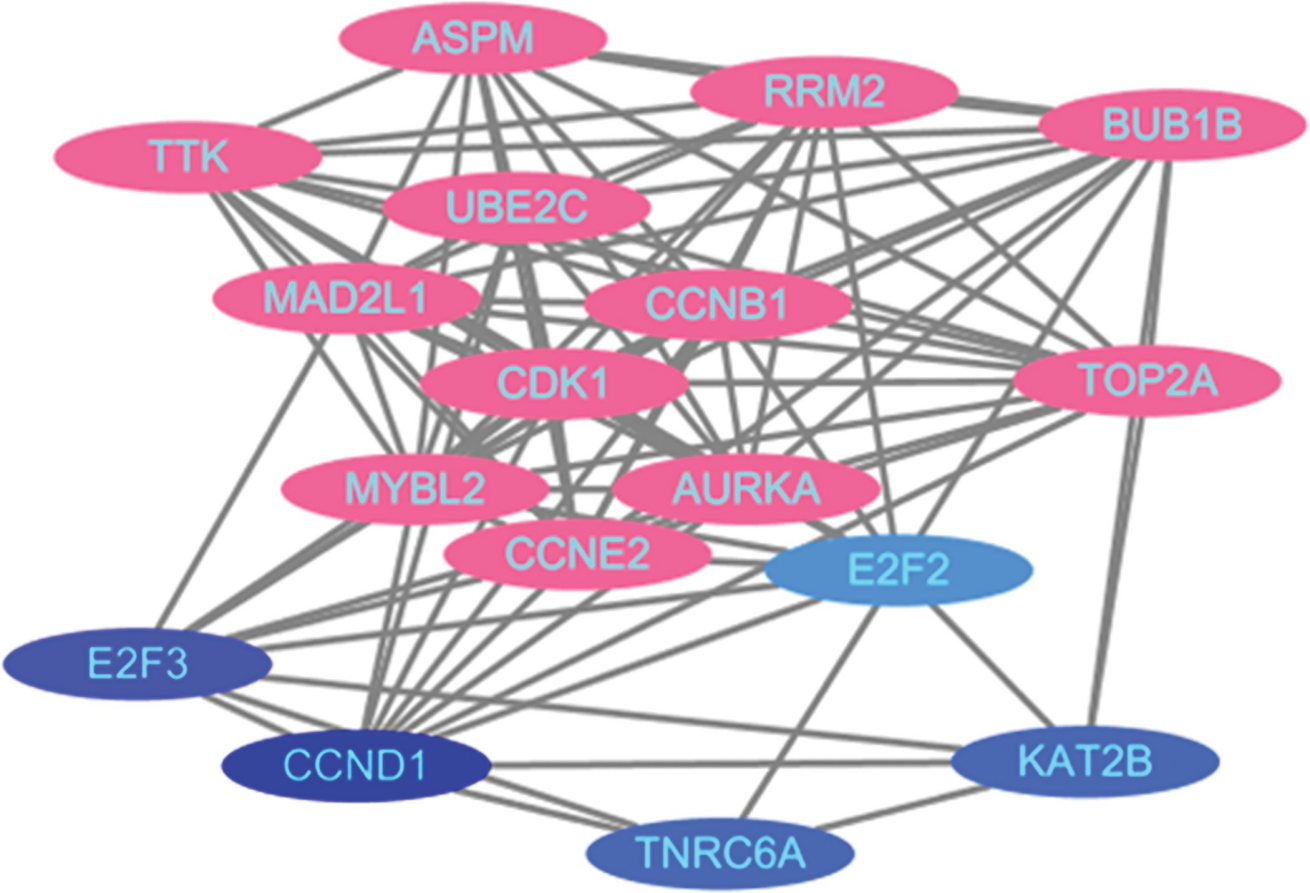
PPI network.

The TSV format file containing PPI data from the STRING database was downloaded and imported into Cytoscape software. Isolated nodes and edges were removed from the network. The PPI network was constructed in Cytoscape software. Key genes were identified using the MCODE plugin. The key genes, namely E2F2, E2F3, and CCND1, were found to be upregulated genes with |log_2_FC| values of 1.283, 1.102, and 2.288, respectively. They had degrees of 4, 58, and 17 in the network. TNRC6A and KAT2B were identified as downregulated genes with |log_2_FC| values of 1.556 and 1.298, respectively. They had degrees of 8 and 5 in the network. In the network visualization, the key genes were represented in blue, and the varying shades of blue indicated the degrees of the nodes (Fig 9).

### 3.7 Regulatory network of key gene circRNA-miRNA-mRNA

**Fig 10:**
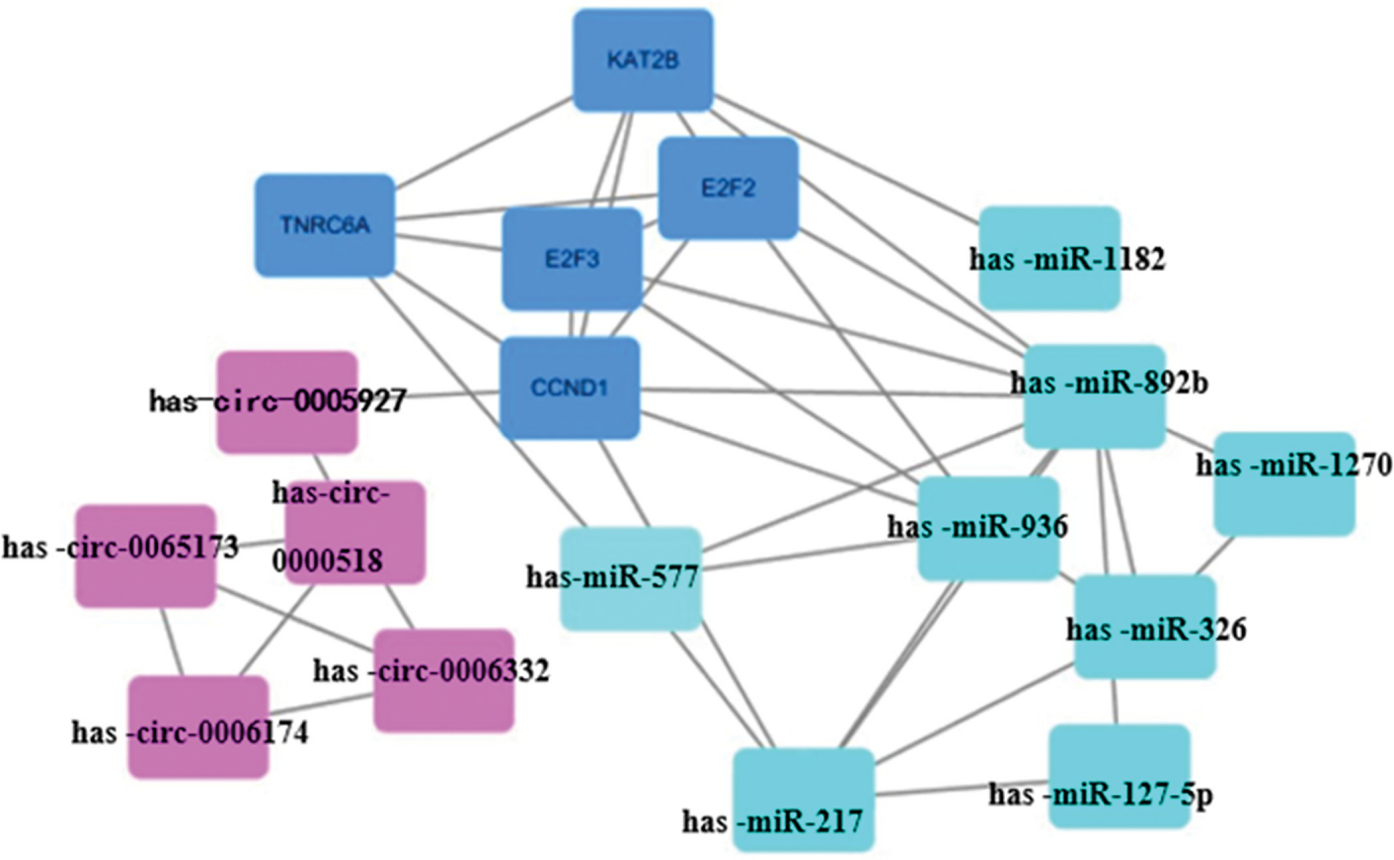
circRNA-miRNA-mRNA regulation network.

Using Cytoscape software, a PPI network was constructed with 5 key genes (E2F2, E2F3, CCND1, TNRC6A, KAT2B), 5 DEcircRNAs (has-circ-0005927, has-circ-0065173, has-circ-0006174, has-circ-0000518, has-circ-0006332), and 8 target miRNAs (has-miR-577, has-miR-217, has-miR-127-5p, has-miR-936, has-miR-892b, has-miR-1182, has-miR-326, has-miR-1270). CCND1 was identified as a key gene in the PPI network. Furthermore, upregulation of has-circ-0087862 was found to weaken the translational inhibition of CCND1 by miR-892b, thereby promoting the expression of CCND1 (Fig 10).

### 3.8 Enrichment analysis results of GO and KEGG

In the g: Profiler database, the set of 96 target DE mRNAs was inputted into the enrichment analysis search box. The source was selected as GO molecular function (MF), GO cellular component (CC), GO biological process (BP), and KEGG. The default significance threshold of adj.P < 0.05 was used, indicating statistical significance.

**Fig 11:**
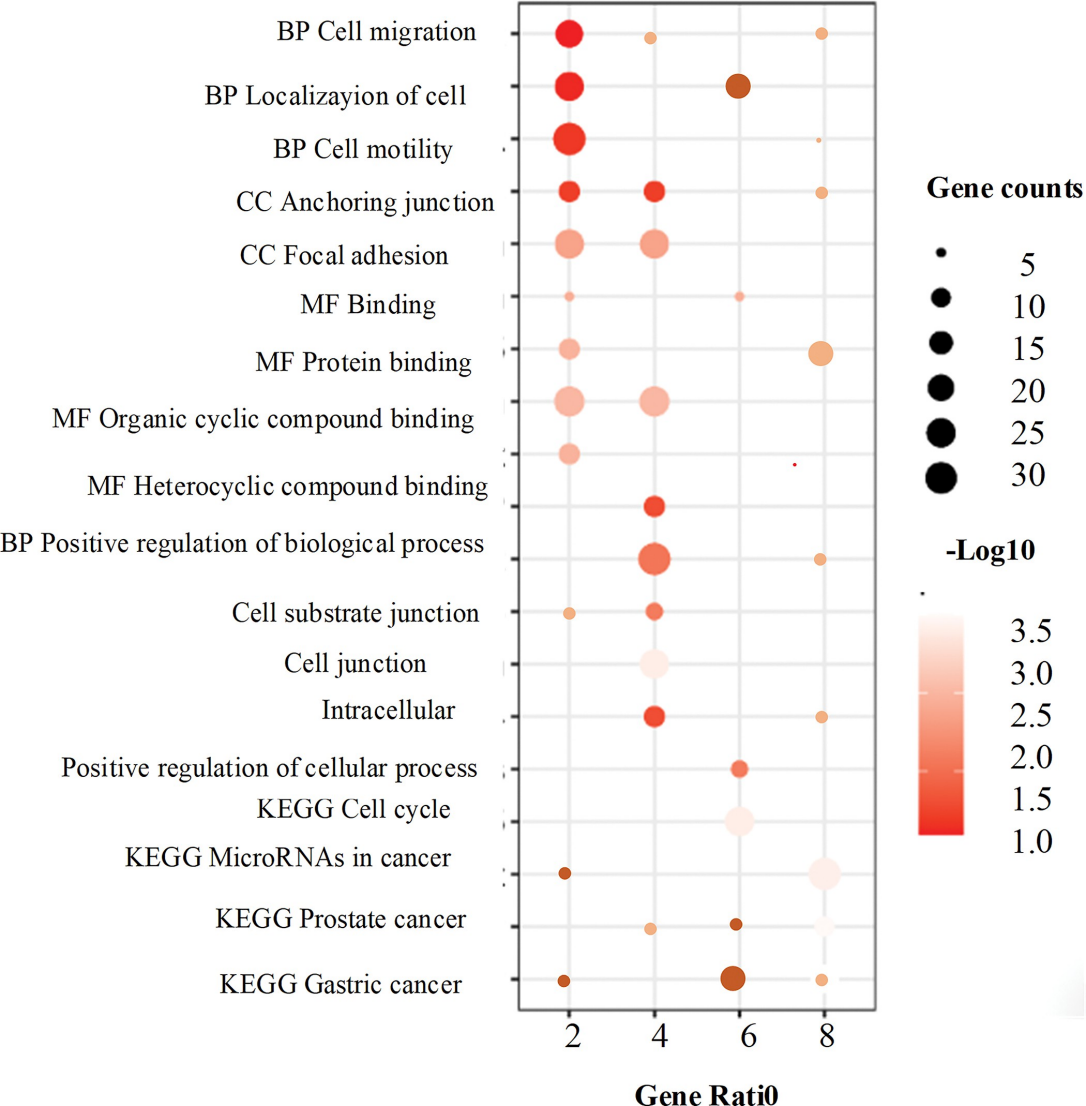
Graphs of GO and KEGG enrichment analysis results.

In the bubble chart of Fig 11, the horizontal axis represents the ratio of the proportion of DEGs belonging to a certain enrichment item to the proportion of all genes belonging to a certain enrichment item. In short, they represent the names of enrichment items. The points in Fig 11 represent the number of genes whose DEGs belong to an enrichment item. The darker the color is, the larger the -Log10 value will be.

Five key genes were identified, and their enrichment in the top 15 categories was analyzed. The results revealed enrichment in several biological processes, including positive regulation of cell regulation, enrichment of cellular components in anchoring junctions, cell junctions, prostate cancer, gastric cancer, hepatitis C, cell cycle, KEGG cell cycle, and binding to heterocyclic compounds. The results also indicated that CCND1 was functionally enriched in protein binding, binding, protein complex binding, and biologically involved in positive regulation of biological processes, cell junctions, and positive regulation of cell processes. The analysis further showed that has-circ-0006332, has-circ-0065173, has-circ-0000518, has-circ-0006174, and has-circ-0005927 were differentially expressed in CRC, suggesting their potential as circRNA diagnostic markers (Table 4).

**Table 4:** Enrichment projects of key genes.

Enrichment	Name	Key gene
Pathway enrichment	Cell cycle	CCND1, E2F2, E2F3
	Cancer microRNA	CCND1, E2F2, E2F3
	Hepatitis C	CCND1, E2F2, E2F3
	Gastric cancer	CCND1, E2F2, E2F3
**Cell component**	Intracellular	E2F2, E2F3, CCND1, TNRC6A, KAT2B
	Cell junction	CCND1
**Biological process**	Positive regulation of cell process	E2F2, E2F3, CCND1, TNRC6A, KAT2B
	Positive regulation of biological process	E2F2, E2F3, CCND1, TNRC6A, KAT2B
**Molecular function**	Protein-containing complex binding	CCND1
	Protein binding	CCND1, E2F2, E2F3, KAT2B
	Heterocyclic compound binding	CCND1, E2F2, E2F3, TNRC6A
	Organic cyclic compound binding	CCND1, E2F2, E2F3, TNRC6A

### 3.9 Effect of overexpression of miR892b on proliferation, migration and invasion of CRC cell SW620

**Fig 12:**
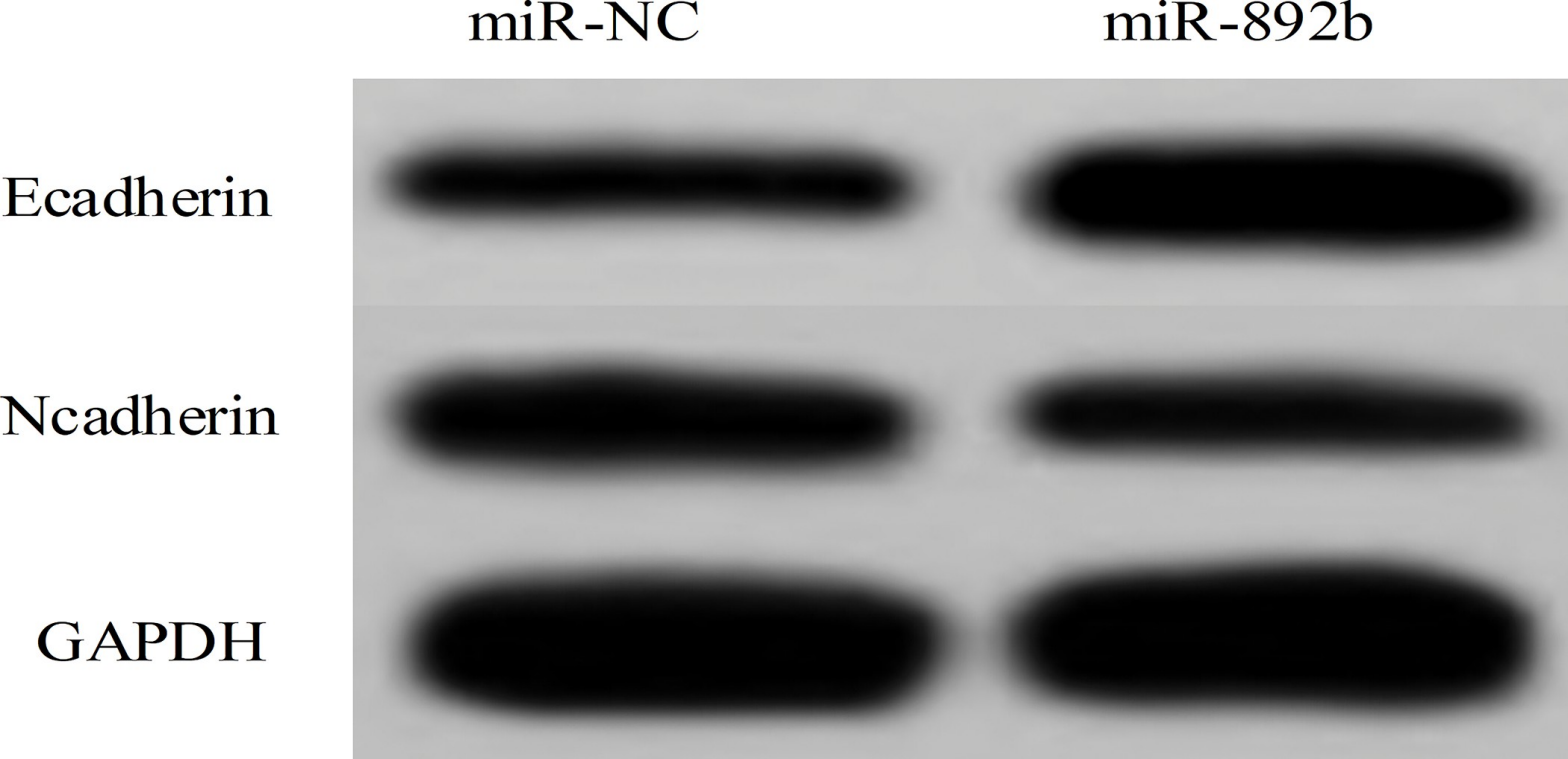
Effect of overexpression of miR892b on the expression of migration and invasion-related proteins in CRC cell SW620.

Fig 12 shows that the expression of miR892b and Ecadherin protein increased significantly compared with miR-NC group (P<0.05), while the number of clones, cell viability, scratch healing rate, number of invasive cells and the expression of Ncadherin protein decreased significantly compared with miR-NC group (P<0.05).

## 4. Discussion

The incidence of CRC in China has been continuously increasing, posing a significant threat to public health. Early diagnosis plays a crucial role in improving outcomes for CRC patients. Several studies have indicated that circRNAs exhibit differential expression in CRC tissues [20, 21]. The identification of novel diagnostic markers can complement existing evaluation techniques and facilitate the early detection of CRC in patients, providing substantial benefits in terms of early diagnosis. Early screening for CRC is essential to improve its prognosis. Liquid biopsies are increasingly being considered for the diagnosis of cancer due to their low invasiveness and high reproducibility. At present, there are many studies on serum diagnostic markers for CRC, and miRNA is one of them [22]. With the development of gene technology, many noncoding RNAs have been identified. As noncoding RNA can be detected in serum,

circRNAs are abundantly expressed in human cells and can also be found in extracellular vesicles. They exhibit resistance to exonucleases and are differentially expressed in CRC, making them potential diagnostic markers. It has been demonstrated that circRNAs can function as ceRNAs by regulating miRNA interactions and controlling miRNA expression at the transcriptional level. In this study, public datasets were utilized to identify the most potent circRNAs involved in CRC regulation and to identify key genes in PPI networks, namely E2F2, E2F3, CCND1, TNRC6A, and KAT2B. ceRNAs are endogenous RNAs that exert biological functions by competitively binding to miRNAs and inhibiting their function. As a type of endogenous competitive RNA, circRNAs competitively bind to miRNAs and indirectly modulate the mRNA targeted by the miRNA, thereby exerting biological functions [23, 24]. Several studies have indicated that miRNAs are central to the regulatory networks, influencing various aspects of cancer cells such as proliferation, apoptosis, invasion, metastasis, and angiogenesis, as well as drug resistance [25]. In CRC, numerous literature reports have demonstrated the endogenous competitive role of circRNAs. CircRNAs act indirectly by competitively binding to miRNAs, thereby attenuating the miRNA-mediated translation inhibition of target mRNAs and exerting biological functions. Depending on the specific target mRNA, circRNAs can exhibit both oncogenic and tumor-suppressive effects in CRC. For instance, upregulated expression of Circ-0000218 competitively binds to miR-139-3p, weakening the translational repression of ras oncogene family members by miR-139-3p, thereby promoting the expression of oncogenes and facilitating invasion and metastasis in CRC, demonstrating its oncogenic role [26, 27]. Cagnoni et al. (2021) [28] reported that the overall ratio of total RNA to free RNA was reduced in CRC compared to normal samples, which could be attributed to the binding of circRNAs to miRNAs. This further underscored the significant role of circRNAs in CRC.

Lin et al. (2019) [29] indicated that miR-202 targeted UHRF1 to inhibit the proliferation and invasion of CRC. Zhu et al. (2015) [30] elaborated that UHRF1 protein was the key for DNA methylation and heterochromatin formation, leading to a reduction in the expression of tumor suppressor genes and promoting tumorigenesis, and verified that miR-9 could inhibit UHRF1 expression and act as a tumor suppressor microRNA in CRC and could be utilized as a prognostic and therapeutic marker for CRC. Khodaii et al. (2022) [31] studied a group of 12 differentially expressed miRs and 11 lncRNAs, as well as 12 genes, in CRC patients. There was drastically increased expression of selected tumor suppressor genes miR and lncRNA, and considerably decreased expression of selected oncomiRs, onco-lncRNAs, and oncogenes following probiotic ingestion compared to placebo. Some network components, such as miR-133b and IGF1 genes, miR-548ac and MSH2 genes, and miR-21 and SMAD4 genes, have strong correlations. The consumption of *Lactobacillus acidophilus* in rectal cancer patients is associated with improved expression of the lncRNA-miR-mRNA network, indicating that the use of the lncRNA-miR-mRNA network can respond to new targets for rectal cancer consumed by *Lactobacillus acidophilus*. Many studies [32] in CRC have demonstrated the endogenous competitive effects of circRNA. CircRNA can attenuate the inhibition of miRNA on mRNA translation and indirectly exert biological functions by competitively binding to miRNA. CircRNAs in CRC can play a role in inhibiting cancer or promoting cancer, mainly based on the different characteristics of mRNAs in CRC [33, 34]. Bachmayr-Heyda et al. (2015) [26] found a decrease in the ratio of overall cyclic RNA to linear RNA in CRC samples from normal tissue samples; that is, circRNA was decreased in CRC samples, which might be due to the binding of circRNA to miRNA, further illustrating that circRNA played a certain key role in CRC. CCND1 is a critical regulator of the cell cycle and exhibits highly conserved periodic fluctuations. Its overexpression can lead to uncontrolled cell proliferation and disease progression. CCND1 plays a significant role in the expression of various cancers. In this study, CCND1 was found to be highly expressed, which holds important implications for the diagnosis and prognosis of CRC.

## 5. Conclusions

Using the GEO database, the datasets GSE126094 and GSE21815 were utilized to identify 5 differentially expressed circRNAs in CRC. The TCGA database was then employed to search for differentially expressed miRNAs in CRC. By mining the TCGA combined with GTEx database, 8 target miRNAs were identified for the differentially expressed mRNAs. Cytoscape software was employed to construct a protein network, which revealed 5 key genes (E2F2, E2F3, CCND1, TNRC6A, KAT2B). CCND1 was identified as a crucial gene in the PPI network. By upregulating has-circ-0087862 and its binding with miR-892b, the translational inhibition of CCND1 by miR-892b was attenuated, leading to an increase in CCND1 expression. There are still many other core genes and mechanisms associated with CRC that require further investigation to provide comprehensive insights into CRC treatment from different perspectives as potential drug targets.

## Supporting information

S1 Data (XLSX)
