# Correction: Bioinformatics analysis-based screening of circRNA gene with mainstream expression trend in colorectal cancer and construction of a coexpression regulatory network

**DOI:** 10.1371/journal.pone.0298924

**Published:** 2024-02-12

**Authors:** Lei Xu, Hongqiang Zhang, Yu Shao, Zan Fu

There are errors in the Funding section. The correct Funding statement is: This work was supported by the National Natural Science Foundation of China (NO.81470881 and NO.82172956), the Jiangsu Commission of Health (LGY2017031 and BRA2015473). The funders had no role in study design, data collection and analysis, decision to publish, or preparation of the manuscript. There was no additional external funding received for this study.
